# Buttock necrosis following uterine artery embolization: A case report

**DOI:** 10.1016/j.ijscr.2022.107833

**Published:** 2022-12-06

**Authors:** Savitri Chandrasekaran, Ramez Antakia, Mojolaoluwa Olugbemi

**Affiliations:** West Suffolk Hospital, Hardwick Ln, Bury St Edmunds IP33 2QZ, United Kingdom

**Keywords:** Fibroid, Embolization, Buttock necrosis, Uterine artery, Skin graft, Case report

## Abstract

**Introduction and importance:**

Fibroid is a very common benign tumor of the uterus for which uterine artery embolization is one of the treatment modalities of choice. Uterine artery embolization (UAE) is a minimally invasive procedure used in the management of fibroids, nowadays mostly performed by interventional radiologists. A rare complication of the procedure is buttock necrosis which has been observed in this case. If not identified at an early stage, it may result in extensive damage to a large surface area of the skin hence this case report is of clinical relevance as it is essential to be aware of the complications of UAE and be cautious.

**Case presentation:**

Our report is regarding a 37-year-old female who presented to the emergency department with a gradually increasing excruciatingly painful lesion on the left buttock 12 days after undergoing a uterine artery embolization. Examination revealed a necrotic lesion involving 40 % surface area of the left buttock.

**Clinical discussion:**

There are a few other such cases reported in literature. All these cases, including ours, prove that buttock necrosis is an established complication which is highly possible following uterine artery embolization. This complication is likely due to reflux of embolic material into gluteal artery.

**Conclusion:**

This case warranted extensive debridement and plastic surgery referral for skin grafting. Although a rare scenario, being a dangerous and distressful one for patients, ability to recognize this complication at an early stage will aid in the management and mental well-being of the patient.

## Highlights

• Uterine artery embolization (UAE) is a minimally invasive procedure used in the management of fibroids.
• Buttock necrosis is a rare complication following uterine artery embolization.
• If ischemic necrosis is not identified at an early stage, it may result in extensive damage to a large surface area of skin.
• This case report is an example of why it is essential to be aware of the complications of UAE and be cautious.

## 1 Introduction

Fibroids, also known as leiomyomas, are common benign tumors of the uterus [1], [2]. They are more prevalent in women of African descent [1]. More often, fibroids are asymptomatic and are incidental findings usually on imaging [1], [3]. If symptomatic, the variety of presentations includes abnormal uterine bleeding, pelvic pain, infertility, back pain or urinary frequency [1], [2], [3]. The symptomatic cases are the ones requiring treatment.

Management of fibroids depends on many factors out of which the age of the patient, symptoms, size and location of the fibroid are the essential ones [1]. While asymptomatic uterine fibroids are usually managed conservatively, treatment modalities for the cases needing intervention are categorized into medical therapies and surgical therapies.

Uterine artery embolization (UAE) is a minimally invasive procedure used in the management of fibroids, nowadays mostly performed by interventional radiologists. This remains an excellent choice in a woman who wants to retain her uterus and potentially avoid more extensive surgery under general anesthesia [4]. However, this procedure has its own risks such as postembolization syndrome, amenorrhea, vaginal discharge, infection and less common complications such as fibroid expulsion, pain, retention of urine and necrosis [5], [6], [7].

## 2 Methods

The SCARE guidelines 2020 was followed to write the case report and we ensured that our work has been written in line with the SCARE 2020 criteria [8].

## 3 Case presentation

A 37-year-old female, with a background of morbid obesity and childhood asthma, presented to the emergency department with a lesion on the left buttock which was gradually increasing in size over one week. It was associated with excruciating pain and generally becoming unwell.

She had no drug history. She lived with her mother, daughter and stepfather. She was a non-smoker and she was mobile independently.

She had undergone a uterine artery embolization performed by an Interventional Radiologist during her recent hospital admission for a large uterine fibroid causing pressure on both ureters, 12 days before she presented acutely to the emergency department. A large volume of embolizing agent (Embozene) up to 2–3 times more than the normal dose was used in view of the challenging arterial puncture due to the patient's high Body Mass Index (BMI). She underwent a uterine artery embolization rather than open surgery because of associated risks with her high BMI (55). The Interventional Radiologist adhered to the local protocols by titrating and increasing the dose gradually till an effective result was achieved. Following the procedure, she became acutely unwell and developed type 2 respiratory failure. After her clinical condition improved, she was discharged on the third day following her procedure.

During the readmission, she complained of developing gradual pain in her left posterior upper thigh and buttock area one day after she underwent uterine artery embolization following which the lesion started. On examining her in the emergency department, a necrotic lesion involving approximately 40 % surface area of left buttock was noted, not extending to the perianal region. The skin around the lesion was tender and mildly erythematous. According to Clavien Dindo classification, this was a stage 3b complication.

Blood tests were done and the inflammatory markers were elevated with a WBC of 15.8 and a CRP of 142. The patient was started on broad spectrum antibiotic cover for this large area of ischaemic necrosis (meropenem + linezolid + clindamycin) and surgical debridement of left gluteal region was performed the following day by a Trust Grade Registrar in General Surgery in the main theatres. She was admitted to the intensive care unit (ITU) following surgery requiring ventilation and vasopressor support. She remained in the ITU for 2 days following which she was extubated successfully and stepped down to the surgical ward.

Wound management was very challenging given the large extent of surgical debridement required, the anatomical location and patient's body habitus requiring initial Vacuum Assisted Closure (VAC) therapy to be carried out in theatre under general anesthesia. Tissue Viability Nurses (TVN) followed up the patient regularly and VAC therapy was applied using Mepitel dressing to protect the wound edges. Once VAC therapy was discontinued, regular wound care and dressing was carried out by the TVN team on the surgical ward.

The patient was very appreciative of the prompt management and decision making with regards to immediate surgical debridement as this was a life-saving procedure. She appreciated the honesty of the surgical team in explaining to her about the consequences and complications secondary to her uterine artery embolization. She understood that her BMI and challenging anatomy contributed to making her interventional radiology procedure more difficult. However, she was obviously traumatized by the prolonged hospital admission and impact of these complications on her quality of life and mental health. The patient's mood deteriorated significantly (given the complexity of her clinical condition and prolonged hospital stay) for which inpatient psychiatry care was integrated in her management.

Tissue specimen sent from the necrotic lesion grew *Staphylococcus aureus* and *Enterococcus faecalis*. Necrotic tissue from gluteal region showed infarcted necrotic skeletal muscle and fat with secondary inflammatory changes. The patient then developed an erythematous rash over her trunk and limbs raising the suspicion of allergic reaction to penicillin, following which antibiotics were switched to vancomycin and ciprofloxacin and the rash gradually subsided.

Plastic surgery consultation was sought for reconstruction of the left gluteal region. Plastic surgeons advised that she needs further wound debridement ± staged wound reconstruction by way of partial direct closure and partial split skin graft in Cambridge university hospital (CUH). Initially, the patient was reluctant to go to CUH but following psychology follow up, encouragement and explanation about the importance of wound reconstruction, she agreed with the plan. The patient was then transferred to CUH for further management of the left buttock wound by the plastic surgeons. Further excision and partial closure by split skin graft was done at CUH.

Two months later, the patient was readmitted with foul smelling discharge from the vagina. A CT abdomen pelvis was done which showed an apparent communication between posterior uterine wall and rectum for which she was managed conservatively. Later, she had a total abdominal hysterectomy done for her fibroid causing back pressure on both ureters and kidneys after extensive discussion at gynecology multi-disciplinary team (MDT).

**Fig. 1:**
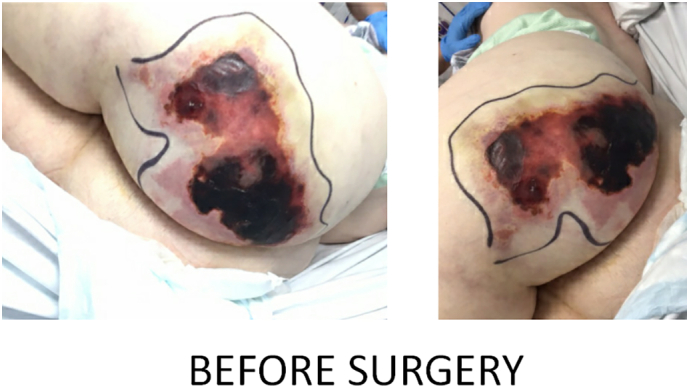
Necrotic lesion prior to surgery.

**Fig. 2:**
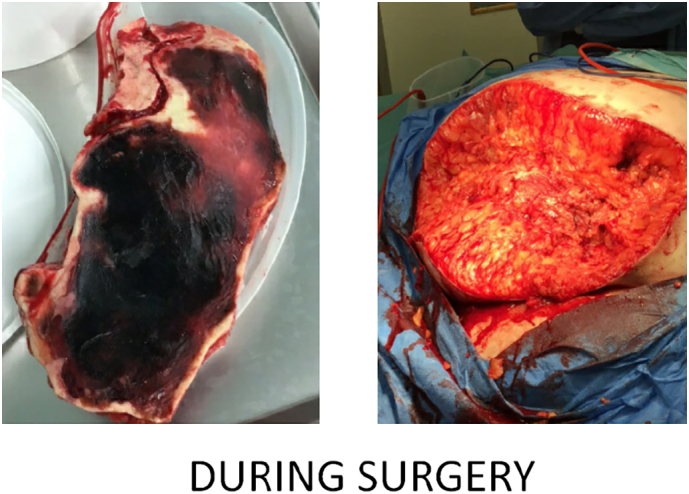
Necrotic lesion during surgery.

**Fig. 3:**
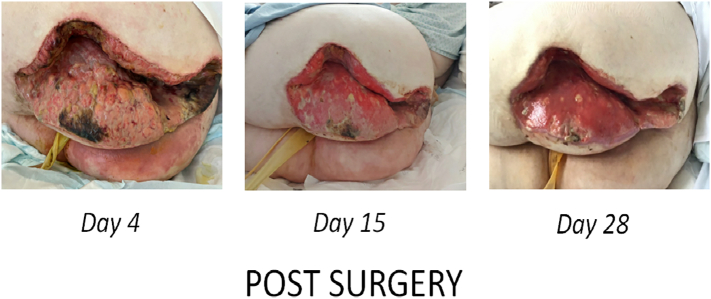
Necrotic lesion after surgery.

The necrotic lesion on the buttock before surgery (Fig. 1), during surgery (Fig. 2) and after surgery (Fig. 3) has been depicted below.

## 4 Discussion

Although uterine artery embolization is being done more commonly nowadays for women with symptomatic fibroid disease, significant and potentially life-threatening complications continue to occur [9]. There have been other reported cases of necrosis following UAE [10], [11], [12], [13]. A case report by Danielle M Dietz et al. described a woman with fibroid who had uterine artery embolization following which she developed two areas of full thickness necrosis on her right buttock [10]. Similarly, Devin D. Smith et al. reported a 39-year-old pregnant lady who developed right buttock necrosis following uterine artery embolization for delayed hysterectomy in placenta percreta [11]. Robin Julve et al. described unexpected unilateral buttock necrosis five days following hypogastric artery embolization for postpartum hemorrhage [12]. The article by Anne-Sophie Didnée, Jr. et al. is yet another example of acute rectal ischemia following uterine artery embolization to treat postpartum hemorrhage [13].

From the above-mentioned cases, it is clear that buttock necrosis is an established complication which is highly possible following uterine artery embolization. The most likely mechanism of this necrosis is the reflux of embolic material from the uterine artery to the inferior gluteal artery [12].

## 5 Conclusion

Although it is a rare scenario, one must keep in mind the complications which can arise from performing a uterine artery embolization. Being aware of potential complications will help in early diagnosis and management of the complication when or if at all it develops. Potentially in challenging cases, a proforma could be developed in order to guide how much embolising agent to be used and whether it would be safer given in smaller doses in more than one session. Furthermore, given potential life-threatening consequences associated with developing ischaemic necrosis, we advise closer follow up in the first week following UAE in an ambulatory setting.

Root cause analysis and case discussion in local morbidity and mortality meeting as part of clinical governance was done. The learning point from this case report is that one must always be aware of the complications while performing a certain procedure and ensure that precautious steps are taken in high-risk cases. In addition, more detailed information could be given to patients on what signs to identify for potential complications and who to contact in case of any deterioration for prompt intervention.

## Consent

Written informed consent was obtained from the patient for publication of this case report and accompanying images. A copy of the written consent is available for review by the Editor-in-Chief of this journal on request.

## Ethical approval

N/A

## Source of funding

None.

## Research registration

N/A.

## Provenance and peer review

Not commissioned, externally peer-reviewed.

## Guarantor

Savitri Chandrasekaran.

## Declaration of competing interest

None.
